# Ultra-processed food consumption and risk of oesophagus, stomach, and pancreatic cancers: a multi case–control study

**DOI:** 10.3389/fnut.2026.1764868

**Published:** 2026-02-26

**Authors:** Laura Torres-Collado, Sandra González-Palacios, Laura María Compañ-Gabucio, Carolina Ojeda-Belokon, Marielisa Gabriela Belisario-Ubeto, Manuela García-de-la-Hera, Alejandro Oncina-Cánovas, Jesús Vioque

**Affiliations:** 1 Unidad de Epidemiología de la Nutrición, Departamento de Salud Pública, Historia de la Ciencia y Ginecología, Universidad Miguel Hernández (UMH), Alicante, Spain; 2 Instituto de Investigación Sanitaria y Biomédica de Alicante (ISABIAL), Departamento de Salud Pública, Hª de la Ciencia y Ginecología, Universidad Miguel Hernández, Alicante, Spain; 3 CIBER Epidemiología y Salud Pública (CIBERESP), Instituto de Salud Carlos III, Madrid, Spain; 4 Departamento de Patología y Cirugía, Universidad Miguel Hernández (UMH), Alicante, Spain

**Keywords:** cancer, oesophagus, pancreas, stomach, ultra-processed food

## Abstract

**Background:**

A high consumption of ultra-processed foods (UPF) has been associated with higher risk of gastrointestinal cancers, although this relationship has been insufficiently investigated. We evaluated the association between UPF consumption and oesophageal, stomach, and pancreatic cancers in a multi-case control study conducted in a Mediterranean area of Spain.

**Methods:**

Data were analysed for 1,218 participants from the PANESOES study, which included incident cases of oesophageal (*n* = 193), stomach (*n* = 412), and pancreatic (*n* = 161) cancers, and 452 controls. Diet was assessed five years before the interview using validated food frequency questionnaire. UPF consumption was estimated in grams/day and as a percentage of total dietary intake using the NOVA classification system and then categorised in tertiles. Multinomial logistic regression was applied to estimate relative risk ratios (RRR) and 95% confidence intervals (CI) adjusting for potential confounders.

**Results:**

UPF consumption in grams/day was positively associated with oesophageal and stomach cancers. Compared with the lowest tertile, the highest tertile of UPF consumption was associated with higher risk of oesophageal cancer (RRR = 2.29; 95% CI: 1.37–3.82) and stomach cancer (RRR = 1.56; 95% CI: 1.08–2.27). For stomach cancer, the highest consumption of ultra-processed dairy products (RRR = 2.10; 95% CI: 1.43–2.82) and sweets/pastries (RRR = 1.76; 95% CI: 1.24–2.50) showed increased risks. Ultra-processed drinks and pre-cooked foods were associated with higher risk of oesophageal cancer. No associations were observed for pancreatic cancer.

**Conclusion:**

Higher UPF consumption was associated with increased risk of oesophageal and stomach cancers, particularly linked to specific UPF categories. Further research is recommended to confirm these results.

## 1 Introduction

Digestive cancers represent a significant global health burden, accounting for a substantial proportion of cancer incidence and mortality worldwide (1). Risk factors for digestive cancers are multifactorial and include genetic predisposition, chronic inflammation, infection (e.g., *Helicobacter pylori* or hepatitis viruses), lifestyle factors such as smoking and alcohol consumption, and particularly, diet quality (2, 3). Growing evidence suggests that certain dietary patterns as well as the consumption of ultra-processed foods (UPF) and low intake of fibre-rich foods, may play a key role in the development of cancer (4, 5).

According to Monteiro et al. (6), UPFs are industrial formulations typically made from substances derived from foods and additives, with little if any whole food added. These products are often characterized nutritionally by elevated levels of total, trans, and saturated fatty acids, salt, and simple sugars, as well as a diminished presence of fibre and micronutrients (6, 7). The growth consumption of these products can result in excessive calorie intake and an imbalance in micro- and macronutrient intake (8). UPF consumption has increased significantly and now constitutes approximately 50 to 60% of the total daily energy intake in medium and high-income countries, replacing traditional eating patterns based on regular freshly prepared meals (9–11). Particularly in Spain, UPFs represented more than 30% of daily energy purchases by 2010 in Spanish households (12). Epidemiological evidence suggests that this extensive consumption and distribution of UPF could be responsible for the global burden and the increasing prevalence of non-communicable diseases such as obesity, cardiovascular disease (CVD), diabetes and various cancers (7, 10, 13–15), which together currently account for approximately 74% of all deaths worldwide (16). In the Spanish population, higher UPF consumption has been associated with increased risk of abdominal obesity (17) and a poorer cardiometabolic profile (18), conditions which have been proposed as potential pathways that may mediate the relationship between UPF consumption and digestive cancer development.

Increasing evidence from prospective cohorts and meta-analyses suggests that high consumption of UPF may be associated with an elevated risk of overall cancer (19). For example, results from large-scale studies such as the NutriNet-Santé cohort (20) and the UK Biobank (21), as well as findings from case–control studies in non-Western populations like Morocco (22), have consistently pointed toward a positive association between UPF consumption and cancer risk. However, most research has focused on overall cancer incidence, and few studies have looked into how UPF consumption relates to specific different types of cancers, particularly within Mediterranean populations.

In this regard, a previous meta-analysis showed that the highest categories of processed food consumption were associated with a 78% increased risk of oesophageal cancer compared with the lowest categories (23). Similar results were observed in the EPIC study, where higher UPF consumption was associated with 24% greater risk of oesophageal cancer (24). In the case of stomach cancer there is limited information, but a case–control study showed an increased risk from higher UPF consumption (25). Finally, in a recent study on pancreatic cancer, a high UPF consumption was found to be associated with an increased risk (14). Nevertheless, the present evidence on the association between UPF consumption and specific digestive cancers (like oesophagus, stomach and pancreas), is very scarce. Thus, we explored the association between UPF consumption and cancers of the oesophagus, stomach, and pancreas in a multi-case control study carried out in a Mediterranean area of Spain.

## 2 Materials and methods

### 2.1 Design and study population

The PANESOES study was a hospital-based, multi-case control study conducted in 9 hospitals in the provinces of Alicante (Hospital General de Alicante, Hospital Universitario de San Juan, Hospital de Elche and Hospital Comarcal de la Vega Baja) and Valencia (Hospital Clínico Universitario, Hospital La Fe, Hospital Dr. Peset, Hospital Arnau de Vilanova and Hospital General de Valencia). The PANESOES study aimed to evaluate the role of lifestyle factors, such as diet, tobacco and alcohol consumption, on the risk of developing three specific types of digestive cancer: oesophagus, stomach, and pancreas. Additional information about the study has been previously outlined (26–29). In short, the PANESOES study sought to enlist 200 participants diagnosed with oesophageal cancer, 400 with stomach cancer, and 200 with pancreatic cancer, along with 450 control participants. The control group was selected through frequency matching to the cases in terms of sex, age (3 categories: 30–59; 60–69; 70–80), and province (Alicante and Valencia). All participants were Spanish-speaking adults, both male and female, ranging from 30 to 80 years old, who were selected after being hospitalized during the period from January 1995 to March 1999. The control group included participants from the same hospital than cases with diagnoses unrelated to the study’s main exposure of interest. These diagnoses were: hernias (34%), degenerative osteoarthritis (21%), fractures/accidents/orthopaedic conditions (19%), appendicitis (6%), and other diagnoses (20%).

**Figure 1:**
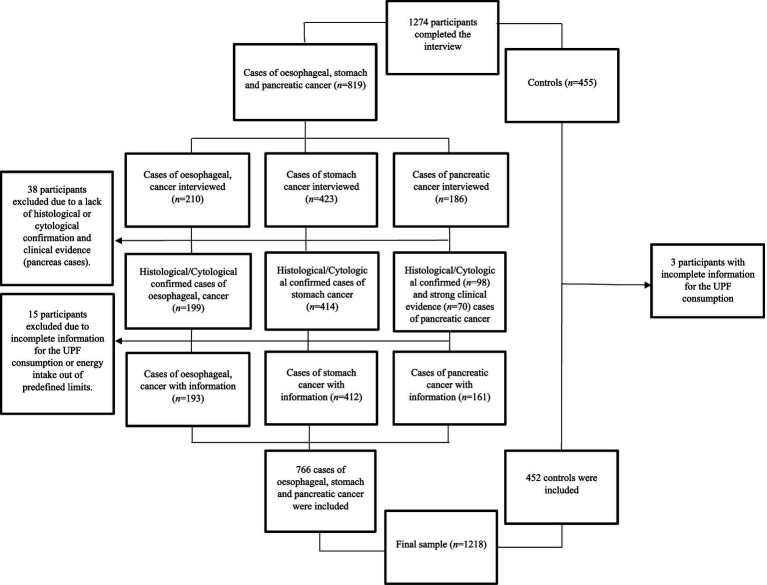
Flow chart describing the selection of the study sample.

In the present study, we included a total of 1,218 participants (828 men and 390 women) with complete information for the variables of interest (Figure 1). The participants included 193 cases of oesophageal cancer, 412 cases of stomach cancer, 161 cases of pancreatic cancer, and 452 control subjects. The diagnoses of all cases of stomach and oesophageal cancers were histologically confirmed from hospital pathology reports, whereas diagnoses of pancreatic cancer were confirmed by two gastroenterologists, based on histological and cytological confirmation, or when lacking, on high clinical evidence. All participants were informed about the study’s objectives and provided written informed consent prior to participation. The research procedures designed for the study were approved by the ethics committees of the hospitals involved and the University Miguel Hernandez (AUT.DSP.JVL.04.21).

### 2.2 Dietary assessment

Information on usual dietary intake was collected using a semi-quantitative food frequency questionnaire (FFQ) which was validated using four 1-week dietary records in a Spanish adult population in the same geographical area (30). The FFQ was based on the Harvard questionnaire adapted for Spain (31), and included 93 food items with nine options for frequency of consumption of each food item, ranging from ‘Never or less than one time per month’ to ‘Six or more times per day’ (30, 32). During the interview, participants were asked how often they consumed the specified standard portion size for each food item over a whole year, referring to the 5 years before the hospital interview. All interviews were conducted by trained personnel following a standardized protocol to ensure consistency in data collection across all participating hospitals.

To evaluate UPF consumption, we used the NOVA classification system, a widely used system for nutritional assessment, which categorises foods based on the purpose, extent, and nature of the processing that they have undergone (33). This system also categorises foods into four groups taking into account the biological, physical, and chemical techniques used for their processing. For this purpose, two trained dietitians independently categorised the 93 food items in the FFQ based on the degree of food processing according to the NOVA system. Any disagreements were resolved by consensus with a third dietitian: Group 1 comprises unprocessed and minimally processed foods like edible parts from plants, animals, fungi, algae, water, and non-alcoholic fermented beverages; Group 2 encompasses processed culinary ingredients used in the preparation, seasoning, and cooking of products from Group 1 such as vegetable oils or spices; Group 3 includes processed foods various fermented beverages like wine or beer; Group 4 comprises ultra-processed, that incorporate items from the first two groups along with palatable foods, primarily pre-prepared, soft drinks and distilled beverages and ready-to-eat products with a high concentration of food additives. The categorization of the FFQ into the four NOVA groups is shown in Table 1. In addition, we subdivided UPF into five subgroups: ultra-processed (UP) dairy products, processed meats, sweets and pastries, pre-cooked food and UP drinks (Table 2).

**Table 1:** Classification of the food frequency questionnaire into the four NOVA groups.

NOVA classification	Food items included
Group 1 *Unprocessed or minimally processed foods*	*Dairy* Whole-milk; skimmed milk; yoghurt. *Eggs, meats, fish, seafood* Chicken eggs; chicken with skin; chicken without skin; beef, pork, lamb; rabbit, quail, duck; liver (beef, pork, chicken); giblets (tripe, brain, gizzard); fish; clam, mussels, oyster; squid, octopus; seafood (shrimps, lobster or similar). *Vegetables and legumes* Spinach (cooked); cabbage, cauliflower, broccoli (cooked); lettuce, endive, chicory; onion; carrot and pumpkin; green beans (cooked); eggplant, zucchini, cucumber; pepper; asparagus; mushroom; cooked legumes (lentils, chickpeas, pinto beans, white beans); cooked peas; garlic. *Fruits* Orange, grapefruit, tangerines; natural orange juice; banana; apple; strawberries; cherries; peach, apricot; fresh figs; watermelon, melon; grapes; nuts (pine nuts, almonds, peanuts, hazelnut). *Bread, grains or others* Home-made potatoes; cooked rice and pasta. *Drinks* Coffee; tea
Group 2 *Processed culinary ingredients*	*Oils and fats* Olive oil; other vegetable oils (sunflower, corn, soy); butter; lard. *Spices* Hot spices (tabasco, chilli pepper); salt; sugar.
Group 3 *Processed foods*	*Dairy* Cheese (ricotta, curd, white, fresh); mature or semi-mature cheese (Manchego). *Meat, fish* Bacon or similar; dry slated fish (cod, anchovies); canned fish (tuna, sardines, herring). *Fruits* Olives; canned fruits in syrup (peach, pear, pineapple). *Bread, grains or others* White bread; wholegrain bread. *Drinks* Wine (white, red, pink); beer; decaffeinated coffee. *Pre-cooked, convenient-food, others* Jam and honey.
Group 4 *Ultra-processed food*	*Dairy* Condensed milk; cheese portions or cream cheese; custard, pudding or similar; ice cream. *Eggs, meats, fish, seafood* Cured ham and processed meats (sausage, salami, bologna); sausages or similar; *pâtés*, foie-gras; hamburgers. *Bread, grains or others* Pretzel; fries; potato chips. *Fat* Margarine. *Sweets and pastries* Biscuits; biscuits with chocolate; croissants, donuts; cupcake, sponge cake; pies, cakes; Spanish *churros*; chocolates, praline; chocolate powder or similar. *Drinks* Brandy, gin, rum, whisky, vodka, spirits; soft drinks with gas (cola, orange soda, fanta or similar); commercial fruit juices. *Pre-cooked, convenient-food, others* *Croquetas*; commercial mayonnaise; packed or canned soups; commercial tomato sauce; fish “sticks” and breadsticks.

**Table 2:** Subgroups of ultra-processed foods.

Ultra-processed food subgroups	Food items included
Dairy products	Condensed milk; custard, pudding or similar; ice cream.
Processed meats	Cured ham and processed meats (sausage, salami, bologna); sausages or similar; *pâtés*, foie-gras; hamburgers.
Sweets and pastries	Margarine, biscuits; biscuits with chocolate; croissants, donuts; cupcake, sponge cake; pies, cakes; Spanish *churros*; chocolates, praline; chocolate powder or similar.
Drinks	Brandy, gin, rum, whisky, vodka, spirits; soft drinks with gas (cola, orange soda, Fanta or similar); commercial fruit juices.
Pre-cooked food	*Croquetas*; commercial mayonnaise; packed or canned soups; commercial tomato sauce; fish “sticks” and breadsticks.

UPF consumption was determined by summing the intake of 25 food items. For each participant, UPF consumption was calculated and expressed in two different ways. First, UPF was calculated as the total number of grams of UPF consumed by each participant, and second UPF was expressed as the percentage of UPF consumption within the participant’s total daily dietary intake, calculated using the formula: ( $\frac{\mathrm{UPF}\;\text{consumption}(g/\mathrm{day})}{\text{Total dietary consumption}(g/\mathrm{day})}*100$ ).

### 2.3 Covariates

We collected participants’ socio-demographic and lifestyle characteristics in personal interviews, which included the three frequency matching variables: age (<60, 60–69 and ≥70years), sex (male or female) and province (Alicante or Valencia). Additionally, we collected information for educational level categorised as less than primary, primary (8 years of schooling) and more than primary, tobacco use (never, former smoker, ≤24 cigarettes/day, >24 cigarettes/day), and group 1, 2 and 3 of NOVA classification (in grams/day). Information on alcohol consumption for each type of beverage was collected separately, i.e., beer, wine, or liquor. The three types of alcohol were further combined to give an overall estimate of the alcohol consumption. One drink was defined as 200 cc of beer, 125 cc of wine, or 50 cc of hard liquor.

### 2.4 Statistical analysis

For description and comparison between cases and controls, we used counts (n) and percentages (%) for categorical variables, while for continuous variables, we used means and standard deviations (SD).

UPF consumption was analysed in tertiles to categorize participants based on their total UPF consumption as ‘low’, ‘medium’, or ‘high’. We performed multinomial logistic regression to estimate relative risk ratios (RRR) and their corresponding 95% confidence intervals (95% CIs) to evaluate the association between UPF consumption in tertiles and the risk of oesophageal, stomach, and pancreatic cancers. The association between UPF consumption and of oesophageal, stomach, and pancreatic cancers was evaluated in two ways, with the UPF consumption expressed in g/day (in tertiles and per 100 g/day of increment) and with the UPF consumption as % of UPF within total daily dietary intake (in tertiles and per 2% of increment). We used the first tertile (low UPF consumption) as the reference category. We used two models: a minimally adjusted model with the frequency-matching variables sex, age, and province, and a more fully adjusted model that included the variables from model 1, further adjusted for factors previously identified in the literature as being associated with these cancers: educational level, smoking habit and groups 1, 2 and 3 of NOVA classification. Lastly, we assessed trends in the RRR across exposure levels and conducted tests for ordinal variables by applying models that used categorical variables as continuous ones.

Statistical significance was established at a threshold of 0.05. All reported *p*-values are derived from two-sided tests. The analyses were conducted using the statistical software STATA, version 16.

## 3 Results

The distribution of cases and controls according to sociodemographic and lifestyle characteristics is shown in Table 3. Educational level was similar for both cases and controls. Alcohol consumption and tobacco smoking were more prevalent in oesophageal cancer than in pancreatic and stomach cancers and controls. In addition, we observed higher energy intake and UPF consumption in oesophageal cancer cases.

**Table 3:** Sociodemographic and lifestyle characteristics among controls and cancer cases (oesophagus, stomach, and pancreas) of the PANESOES study (*n* = 1,218).

Variables	Controls	Cases		
		Oesophagus	Stomach	Pancreas
*n* (%)	452 (37.1)	193 (15.8)	412 (33.8)	161 (13.2)
Age, *n* (%)				
30–59 years	148 (32.7)	79 (40.9)	117 (28.4)	39 (24.2)
60–69 years	166 (36.7)	78 (40.4)	129 (31.3)	50 (31.1)
70–80 years	138 (30.5)	36 (18.6)	166 (40.3)	72 (44.7)
Sex, n (%)				
Male	282 (62.4)	178 (92.2)	270 (65.5)	98 (60.9)
Female	170 (37.6)	15 (7.8)	142 (34.5)	63 (39.1)
Province, *n* (%)				
Valencia	314 (69.5)	151 (78.2)	281 (68.2)	102 (63.3)
Alicante	138 (30.5)	42 (21.8)	131 (31.8)	59 (36.7)
Educational level, *n* (%)				
< Primary	244 (54.0)	110 (57.0)	249 (60.4)	89 (55.3)
Primary	171 (37.8)	65 (33.7)	128 (31.1)	53 (32.9)
> Primary	37 (8.2)	18 (9.3)	35 (8.5)	19 (11.8)
Alcohol consumption, *n* (%)				
Never	181 (40.1)	17 (8.8)	145 (35.2)	54 (33.5)
1–24 g/day	162 (35.8)	35 (18.1)	131 (31.8)	56 (34.7)
25–49 g/day	50 (11.0)	23 (11.9)	64 (15.5)	14 (8.7)
50–99 g/day	41 (9.1)	51 (26.4)	47 (11.4)	25 (15.5)
> 99 g/day	18 (4.0)	67 (34.7)	25 (6.1)	12 (7.4)
Tobacco use, *n* (%)				
Never	216 (47.8)	22 (11.4)	172 (42.0)	70 (43.5)
Former	117 (25.9)	53 (27.5)	91 (22.2)	34 (21.1)
Current ≤ 24 c/day	87 (19.2)	57 (29.5)	106 (25.8)	36 (22.4)
Current > 24 c/day	32 (7.1)	61 (31.6)	41 (10.0)	21 (13.0)
Energy intake (kcal/day), mean (SD)	1800.8 (620)	2274.3 (821)	1996.8 (662)	1934.4 (741)
Group 1 of NOVA (g/day), mean (SD)	1233.5 (429)	1256 (495)	1267 (494)	1285.7 (493)
Group 2 of NOVA (g/day), mean (SD)	25.1 (23.7)	24.4 (19.6)	27.1 (21.5)	27.1 (23.3)
Group 3 of NOVA (g/day), mean (SD)	400 (348)	833 (592)	500.5 (444)	492.9 (467)
Group 4 of NOVA (g/day), mean (SD)	130.2 (134)	232.8 (273)	157.9 (161)	143.1 (159)
UP dairy consumption (g/day), mean (SD)	8.8 (15.5)	11.6 (24.1)	14.4 (27.1)	9.4 (14.5)
Processed meat consumption (g/day), mean (SD)	24.0 (14.1)	26.3 (16.7)	26.2 (16.2)	26.4 (16.5)
UP sweet and pastries consumption (g/day), mean (SD)	28.0 (31.2)	24.1 (28.4)	34.4 (32.6)	34.0 (41.6)
UP drinks consumption (g/day), mean (SD)	53.4 (122.5)	144.8 (260.9)	64.6 (139.6)	53.5 (126.9)
Pre-cooked food (g/day), mean (SD)	16.0 (24.9)	26.0 (42.1)	18.2 (26.4)	19.7 (30.0)

SD, Standard Deviation; g, grams; c/day, cigarettes per day; Kcal, kilocalories, UP, Ultra-processed.

The association between total consumption of UPF and risk of oesophageal, stomach, and pancreatic cancers is presented in Table 4. In multivariate analysis, a higher UPF consumption was associated with higher risk of oesophagus and stomach cancer. Compared with the lowest tertile of UPF consumption, the medium and highest tertiles of UPF consumption were associated with higher risk of oesophagus cancer, RRR = 1.85 (1.13–3.04) and RRR = 2.29 (1.37–3.82), respectively. For every 100 grams of UPF consumption, the risk of oesophagus cancer increased by a 25%. Similarly, compared with the lowest tertile of UPF consumption, the medium and highest tertiles were associated with higher risk of stomach cancer, RRR = 1.58 (1.13–2.21) and RRR = 1.56 (1.08–2.27), respectively. In addition, a 100-gram increase in UPF consumption was associated with 13% higher risk of stomach cancer. No associations were observed for pancreatic cancer.

**Table 4:** Association between ultra-processed food (UPF) consumption in g/day among oesophagus, stomach and pancreas cancers, and controls of the PANESOES study (*n* = 1,218).

UPF consumption					
	Low intake	Medium intake	High intake		Per 100 g/day increase
	(< 74.3 g)	(74.3–148.0 g)	(> 148 g)		
	RRR (IC 95%)	RRR (IC 95%)	RRR (IC 95%)	p-trend	RRR (IC 95%)
Oesophagus, n	39	64	90		193
Model 1	Ref	2.05 (1.28–3.28)	3.07 (1.91–4.94)	<0.001	1.31 (1.17–1.45)
Model 2	Ref	1.85 (1.13–3.04)	2.29 (1.37–3.82)	0.001	1.25 (1.12–1.40)
Stomach, n	122	150	90		412
Model 1	Ref	1.68 (1.20–2.33)	1.80 (1.25–2.58)	0.001	1.16 (1.05–1.29)
Model 2	Ref	1.58 (1.13–2.21)	1.56 (1.08–2.27)	0.012	1.13 (1.02–1.25)
Pancreas, n	60	54	47		161
Model 1	Ref	1.19 (0.77–1.85)	1.14 (0.70–1.85)	0.702	1.09 (0.95–1.26)
Model 2	Ref	1.04 (0.66–1.62)	0.90 (0.54–1.48)	0.619	1.04 (0.90–1.20)

Model 1: adjusted by age, sex and province. Model 2: Model 1 plus educational level, smoking habit and the groups 1, 2 and 3 of NOVA.

UPF, ultra-processed food; g, grams; RRR; relative risk ratios.

When we evaluated the association between UPF consumption as percentage of total food consumption, we also observed a higher risk for oesophageal and stomach cancers (Supplementary Table 1). Compared with the lowest tertile of UPF consumption, the highest tertile was linked to a 68% higher risk of oesophageal cancer. For every 2% increment of UPF consumption, the risk of oesophagus cancer increased by an 11% and the risk of stomach cancer increased by a 6%. No association was observed for pancreatic cancer (Supplementary Table 1).

The associations between the different subgroups of UPF in total grams per day and oesophageal, stomach, and pancreatic cancers are presented in Table 5. For UP dairy products, the consumption of 100g/day was significantly associated with a higher risk of oesophageal cancer, RRR = 3.08 (1.16–8.13), although no evidence of trend was observed by tertiles of consumption. For stomach cancer, a significant trend was observed by tertiles of consumption (p-trend <0.001), medium and highest tertiles showed higher risk of stomach cancer, RRR = 1.44 (1.03–2.03) and RRR = 2.10 (1.43–2.82), respectively. Moreover, each additional 100g/day of consumption was significantly associated with a higher risk, RRR = 4.13 (1.85–9.20). Similarly, the consumption of sweets and pastries was positively associated with stomach cancer, compared to lowest tertile, those in the medium and highest tertiles showed higher risks, RRR = 1.79 (1.27–2.51) and RRR = 1.76 (1.24–2.50), respectively (p-trend = 0.002); the consumption of 100 g/day of sweets and pastries was associated with higher risk of stomach cancer, RRR = 1.76 (1.14–2.72). Regarding UP drinks, the highest tertile of consumption was associated with a higher risk of oesophageal cancer, RRR = 1.81 (1.13–2.90), with a significant positive trend (p-trend = 0.021); in addition, every 100 g/day of UP drinks increased in consumption was associated with a 21% higher risk of oesophageal cancer. Finally, consumption of pre-cooked foods also increased the risk of oesophageal cancer, every 100 g/day of consumption was associated with higher risk, RRR = 2.83 (1.54–5.19). No association was observed for processed meat consumption and any type of digestive cancer. Similar results were observed when we assessed the effect of UPF subgroups as percentage of total food consumption (Supplementary Table 2).

**Table 5:** Association between main UPF subgroups consumption among controls and cancer cases (oesophagus, stomach, and pancreas) of the PANESOES study (*n* = 1,218).

UPF Subgroups consumption					
	RRR (IC 95%)	RRR (IC 95%)	RRR (IC 95%)	p-trend	RRR (IC 95%)
UP dairy products	Low intake	Medium intake	High intake		100 g increase
	0 g	0.1–8.4 g	> 8.4 g		
Oesophagus, *n*	100	39	54		193
Model 1	Ref	0.67 (0.43–1.04)	1.01 (0.67–1.54)	0.886	2.41 (0.93–6.26)
Model 2	Ref	0.68 (0.43–1.10)	1.10 (0.70–1.73)	0.874	3.08 (1.16–8.13)
Stomach, n	144	116	152		412
Model 1	Ref	1.43 (1.03–2.00)	2.08 (1.50–2.90)	<0.001	4.24 (1.93–9.32)
Model 2	Ref	1.44 (1.03–2.03)	2.10 (1.43–2.82)	<0.001	4.13 (1.85–9.20)
Pancreas, n	68	46	47		161
Model 1	Ref	1.21 (0.78–1.88)	1.29 (0.83–2.03)	0.200	1.19 (0.36–3.91)
Model 2	Ref	1.21 (0.78–1.89)	1.18 (0.74–1.87)	0.473	0.98 (0.30–3.39)
Processed Meats	Low intake	Medium intake	High intake	p-trend	100 g increase
	< 17.6g	17.6–28.6g	> 28.6g		
Oesophagus, *n*	67	79	47		
Model 1	Ref	0.84 (0.56–1.26)	0.91 (0.57–1.46)	0.606	1.24 (0.38–4.03)
Model 2	Ref	0.87 (0.57–1.35)	0.70 (0.42–1.17)	0.453	0.55 (0.15–1.97)
Stomach, *n*	134	175	103		
Model 1	Ref	1.08 (0.79–1.49)	1.43 (0.98–2.07)	0.068	2.87 (1.16–7.06)
Model 2	Ref	1.09 (0.80–1.50)	1.24 (0.85–1.83)	0.220	2.03 (0.80–5.17)
Pancreas, *n*	52	72	37		
Model 1	Ref	1.24 (0.81–1.88)	1.43 (0.86–2.38)	0.187	3.76 (1.19–11.91)
Model 2	Ref	1.21 (0.80–1.86)	1.12 (0.80–1.86)	0.521	2.16 (0.65–7.12)
Sweets and pastries	Low intake	Medium intake	High intake	p-trend	100 g increase
	< 7.9 g	7.9–35.8g	> 35.8g		
Oesophagus, *n*	83	58	52		193
Model 1	Ref	0.92 (0.61–1.40)	0.79 (0.52–1.22)	0.369	0.65 (0.35–1.20)
Model 2	Ref	0.93 (0.57–1.45)	0.95 (0.60–1.51)	0.571	0.81 (0.41–1.56)
Stomach, *n*	103	156	153		412
Model 1	Ref	1.79 (1.28–2.50)	1.75 (1.24–2.46)	0.001	1.74 (1.14–2.67)
Model 2	Ref	1.79 (1.27–2.51)	1.76 (1.24–2.50)	0.002	1.76 (1.14–2.72)
Pancreas, *n*	50	53	58		161
Model 1	Ref	1.20 (0.76–1.89)	1.29 (0.82–2.02)	0.254	1.58 (0.91–2.74)
Model 2	Ref	1.20 (0.76–1.90)	1.28 (0.80–2.03)	0.326	1.59 (0.90–2.81)
UP drinks	Low intake	Medium intake	High intake	p-trend	100 g increase
	0 g	0.1–35.7g	> 35.7 g		
Oesophagus, *n*	50	37	106		193
Model 1	Ref	1.05 (0.64–1.74)	2.95 (1.90–4.57)	<0.001	1.27 (1.14–1.41)
Model 2	Ref	0.92 (0.55–1.56)	1.81 (1.13–2.90)	0.021	1.21 (1.08–1.36)
Stomach, *n*	199	85	128		
Model 1	Ref	0.80 (0.57–1.14)	1.19 (0.85–1.67)	0.406	1.08 (0.97–1.21)
Model 2	Ref	0.74 (0.52–1.07)	1.00 (0.70–1.42)	0.996	1.06 (0.95–1.18)
Pancreas, *n*	79	37	45		
Model 1	Ref	0.91 (0.57–1.45)	1.10 (0.69–1.75)	0.952	1.02 (0.89–1.20)
Model 2	Ref	0.83 (0.52–1.34)	0.87 (0.53–1.40)	0.381	0.98 (0.84–1.16)
Pre-cooked food	Low intake	Medium intake	High intake	p-trend	100 g increase
	< 5.4g	5.4–15.0g	> 15.0g		
Oesophagus, *n*	684	53	76		
Model 1	Ref	1.22 (0.78–1.90)	1.82 (1.18–2.82)	0.005	2.95 (1.64–2.29)
Model 2	Ref	1.04 (0.64–1.67)	1.46 (0.91–2.35)	0.120	2.83 (1.54–5.19)
Stomach, *n*	142	140	130		
Model 1	Ref	1.50 (1.07–2.09)	1.23 (0.87–1.74)	0.223	1.40 (0.80–2.43)
Model 2	Ref	1.31 (0.93–1.84)	1.06 (0.74–1.53)	0.733	1.25 (0.72–2.17)
Pancreas, *n*	60	41	60		
Model 1	Ref	1.02 (0.63–1.62)	1.25 (0.79–1.97)	0.374	1.58 (0.80–3.13)
Model 2	Ref	1.01 (0.63–1.63)	1.13 (0.71–1.80)	0.993	1.37 (0.69–2.71)

Model 1: adjusted by age, sex and province. Model 2: Model 1 plus educational level, smoking habit and the groups 1, 2 and 3 of NOVA.

UPF, ultra-processed food; g, grams; RRR; relative risk ratios.

## 4 Discussion

In this multi-case control study, we observed that higher consumption of UPF was associated with an increased risk of oesophageal and stomach cancers. Regarding subgroups of UPF, the consumption of UP dairy products was associated with greater risk of oesophageal and stomach cancers; the consumption of sweets and pastries was associated with stomach cancer and the consumption of UP drinks and pre-cooked foods was linked to an increased risk of oesophageal cancer. No associations were found between UPF consumption and the risk of pancreatic cancer.

A previous systematic umbrella review based on existing meta-analyses concluded that higher UPF consumption was associated with an increased risk of overall cancer (34). The most recent evidence based on observational studies have shown a positive association between UPF consumption and overall cancer mortality (21, 35), although others studies have found no association (36–38). The association found in our study for oesophageal and stomach cancer would be in line with the present general evidence although it is based on two cancer sites only.

However, our results for oesophageal cancer are consistent with those reported in other studies. In a population-based case–control study in China (39), the highest quartile of UPF consumption was linked to an almost 3-fold increased risk of developing oesophageal cancer. Similarly, in the EPIC multi-centre prospective cohort study in Europe, the increment of 10% g/day of UPF consumption was associated with a 24% increase in the risk of oesophageal cancer (24). In the same line, Chang et al. (21) in the UK Biobank prospective cohort study showed that per 10% increment in UPF consumption in the total diet, the risk of oesophageal cancer increased by 8%, although this association was not statistically significant.

When we explored the association between the subgroups of UPF and oesophageal cancer risk, we found significant positive associations with UP dairy products, drinks and pre-cooked foods. In the study by Navarro Silvera et al. (40), the consumption of high-fat dairy, mostly UP dairy foods, including cheese or cheese spreads, ice cream, milkshakes, puddings, custard, flan or milk-based beverages, was associated with an increased risk of both oesophageal adenocarcinoma and squamous cell carcinoma; however, other epidemiological studies did not find associations between specific categories of dairy products and the risk of developing oesophageal cancer (41, 42). In contrast, recent epidemiological evidence suggests a positive association between the consumption of UP drinks like sugar-sweetened beverages (SSB) or alcoholic drinks and oesophageal cancer. In a pooled analysis of two case–control studies in US (43), the SSB consumption was associated with a 55% increase in oesophageal cancer risk. In addition, in a dose–response meta-analysis by Yu et al. (44), each additional 12.5 g/day of ethanol consumption was associated with a 33% increase in oesophageal cancer risk, with higher risks for spirits and liquors. Similarly, in a meta-analysis of 106 cohort studies (45), the association between alcohol intake and oesophageal cancer was evident even for low levels of intake. The positive association between pre-cooked foods and oesophageal cancer we found is consistent with that reported by Yan et al. in a recent meta-analysis (23).

Previous studies have shown an increased risk for gastrointestinal cancer related to UPF consumption (21, 46), but few studies have specifically explored stomach cancer. In our study, we observed that high UPF consumption was associated with a 56% higher risk of stomach cancer. In a recent meta-analysis of five prospective cohort studies (4) the highest UPF consumption was significantly associated with an increased risk of non-cardia gastric cancer, while no association was found with gastric cardia cancer. Similarly, a prospective cohort study within the UK Biobank (21) found that the highest level of UPF consumption was linked to a greater risk of non-cardia gastric cancer, while an inverse effect was found between high UPF consumption and gastric cardia cancer, although none of these associations were statistically significant. When we evaluated specific associations between UPF subgroups, we found that the consumption of UP dairy products and sweets and pastries were associated with a higher risk of stomach cancer. These results are consistent with those reported by Tayyem et al. (47), who observed an increased risk of stomach cancer among participants with higher consumption of UP dairy products. Furthermore, in a previous analysis of the PANESOES study population (48), a high consumption of UP dairy products was associated with an 85% higher risk of stomach cancer. In addition, Peres et al. (25), carried out a study in Brazilian population and found that medium and high intake of sweets increased the risk of stomach cancer by 74 and 125%, respectively. Similarly, Almahri et al. (49), showed that the intake of sweets such as candies and biscuits was significantly associated with an 87% increased risk of stomach cancer.

Finally, regarding the effect of UPF consumption on pancreatic cancer, we did not observe any association. In the EPIC study, the UPF consumption showed evidence of a certain trend, although it did not reach significance (50). However, in a population-based cohort study of 98,265 American adults (14), a high consumption of UPF was associated with an increased risk of pancreatic cancer; participants in the highest quartile of UPF consumption had 47% higher risk of pancreatic cancer compared with the lowest quartile. To the best of our knowledge, no prior studies have specifically examined the relationship between subgroups of UPF and pancreatic cancer. Unlike our findings for the other cancers, no significant associations were found between the consumption of UPF subgroups and the risk of pancreatic cancer.

With respect to potential mechanisms that may underlie the observed positive association between UPF and cancer we should consider the obesogenic properties of some UPF, as well as the exposure to food additives and contaminants (11). In addition, other factors may play a role, such as the relationship between UPF consumption and a poor overall diet quality with excessive calorie intake, primarily due to the high energy density and low satiety often associated with these foods (8). Some studies have shown that diets high in UPF tend to contain significant levels of trans fatty acids, salt, and/or sugars as well as lower content in potassium, protein and fibre, which have been associated with higher prevalence of non-communicable diseases in observational studies (51). Another potential mechanism may relate to the link between higher consumption of UPF and obesity (52), a known risk factor for overall and specific cancers like breast, colorectal and pancreatic cancer (19). Food contaminants found in UPF like neo-formed compounds that arise during their chemical, biological, and physical transformations, could also represent a potential mechanism for increasing the risk of cancer; in this sense, UPF typically contains neo-formed processing contaminants (e.g., trans fatty acids) that may be positively associated with cancer risk due to high-heat processing (53, 54). It has also been suggested that several contaminants from food packaging may be present in UPF. For example, increased urinary concentrations of bisphenol and phthalates were observed in a young U. S. population with high UPF consumption, which could suggest that UPF packaging might be one of the links potentially contributing to adverse health outcomes, including cancer (55). However, further research is required to confirm whether additives, packaging compounds, or heat-produced neo-formed contaminants can contribute to cancer development, since the observational nature of our study cannot establish causality.

We acknowledge limitations in our study. As in other case–control studies, our study is more susceptible to certain types of bias, such as selection bias. However, in this regard, it is important to note that the participation rate of cases and controls was similar. Another limitation inherent to the case–control design is recall bias. In this sense, although dietary intake data were collected during hospital interviews, they referred to the five years prior to diagnosis making more unlikely that diet be a consequence of the disease when the interview was done. In addition, the study sample was limited (especially for oesophageal and pancreatic cancers), which hinder the possibility of exploring associations by histological subtype. The observational nature of our study limits the possibility of inferring causal relationships, making it necessary to conduct further studies with larger samples with a long follow-up period, although these studies may be less feasible. Moreover, despite performing multivariate analyses that included relevant known risk factors such as tobacco and alcohol consumption, the possibility of residual confounding remains.

Our study also has several strengths. Firstly, we used the NOVA classification system, which is a well-established and widely used system in the literature and allowed us to specifically and accurately examine the association between UPF and digestive cancer. Secondly, our dietary assessment tool has been previously validated (30). Thirdly, our findings align with those reported in the existing literature regarding the harmful effects of UPF consumption. Nevertheless, our results are novel because we explore the above-mentioned effect on three different gastrointestinal cancers which have been understudied despite being highly relevant in public health. Finally, although our study was hospital-based, our findings may be generalizable to other populations with similar characteristics, as cases and controls were recruited from public referral hospitals within the Spanish National Health System, which provides universal and free access to healthcare for the entire population.

Our study suggests that higher UPF consumption is associated with higher risk of oesophageal and stomach cancers, and that the UPF subgroups associated were UP dairy products, sweets and pastries, alcoholic drinks and pre-cooked foods. No association was observed for pancreatic cancer. Further studies are needed to confirm these results.

## Data Availability

The data analyzed in this study is subject to the following licenses/restrictions: the raw data supporting the conclusions of this article will be made available by the authors without undue reservation. Requests to access these datasets should be directed to Jesús Vioque, vioque@umh.es.
