# Supplementary material for: Ultra-processed food consumption and risk of oesophagus, stomach, and pancreatic cancers: a multi case–control study
Source: Front Nutr. 2026 Feb 26;13:1764868. doi: 10.3389/fnut.2026.1764868 (PMC12979122; doi:10.3389/fnut.2026.1764868)
Supplement: Supplementary file 1 [file Table_1.DOCX]

| **Supplementary table 1.** Association between ultra-processed food (UPF) consumption (% of total daily intake in g/day) among controls and cancer cases (oesophagus, stomach, and pancreas) of the PANESOES study (n=1,218). | | | | | |
| --- | --- | --- | --- | --- | --- |
|  | **UPF consumption** | | |  |  |
|  | **Low intake** | **Medium intake** | **High intake** |  | **2 % increase** |
|  | (< 4.3%) | (4.3-7.7%) | (> 7.7%) |  |  |
|  | **RRR**  **(IC 95%)** | **RRR**  **(IC 95%)** | **RRR**  **(IC 95%)** | **p-trend** | **RRR**  **(IC 95%)** |
| **Oesophagus**, n | 55 | 61 | 77 |  | 193 |
| Model 1 | Ref | 1.29 (0.83-2.01) | 1.63 (1.05-2.52) | 0.073 | 1.09 (1.04-1.15) |
| Model 2 | Ref | 1.49 (0.93-2.40) | 1.68 (1.05-2.69) | 0.079 | 1.11 (1.05-1.17) |
| **Stomach**, n | 130 | 143 | 139 |  | 412 |
| Model 1 | Ref | 1.18 (0.84-1.64) | 1.21 (0.86-1.64) | 0.291 | 1.05 (1.01-1.10) |
| Model 2 | Ref | 1.22 (0.87-1.71) | 1.22 (0.86-1.73) | 0.228 | 1.06 (1.01-1.11) |
| **Pancreas**, n | 60 | 54 | 47 |  | 161 |
| Model 1 | Ref | 0.91 (0.58-1.40) | 0.82 (0.52-1.30) | 0.316 | 0.99 (0.93-1.06) |
| Model 2 | Ref | 0.92 (0.59-1.44) | 0.79 (0.49-1.27) | 0.284 | 1.00 (0.93-1.07) |
| Model1: adjusted by age, sex and province. Model 2: Model 1 plus educational level, smoking habit, beer and wine consumption.  Abbreviations: UPF, ultra-processed food; RRR; relative risk ratios. | | | | | |

Supplementary Material

| **Supplementary table 2.** Association between main ultra-processed food (UPF) subgroups consumption among controls and cancer cases (oesophagus, stomach, and pancreas) of the PANESOES study (n=1,218). | | | | | |
| --- | --- | --- | --- | --- | --- |
|  | **UPF consumption** | | |  |  |
| **UP dairy products** | **Low intake** | **Medium intake** | **High intake** |  | **2 % increase** |
|  | 0% | 0.1-9.2% | >9.2% |  |  |
|  | **RRR (IC 95%)** | **RRR (IC 95%)** | **RRR (IC 95%)** | **p-trend** | **RRR (IC 95%)** |
| **Oesophagus**, n | 100 | 46 | 47 |  | 193 |
| Model 1 | Ref | 0.91 (0.59-1.42) | 0.76 (0.50-1.16) | 0.313 | 0.99 (0.96-1.02) |
| Model 2 | Ref | 0.82 (0.51-1.58) | 1.01 (0.65-1.58) | 0.824 | 1.02 (0.98-1.05) |
| **Stomach**, n | 144 | 98 | 170 |  | 412 |
| Model 1 | Ref | 1.57 (1.10-2.25) | 1.84 (1.35-2.51) | <0.001 | 1.04 (1.01-1.06) |
| Model 2 | Ref | 1.56 (1.08-2.24) | 1.92 (1.40-2.64) | <0.001 | 1.04 (1.02-1.06) |
| **Pancreas**, n | 68 | 44 | 49 |  | 161 |
| Model 1 | Ref | 1.51 (0.95-2.39) | 1.09 (0.71-1.66) | 0.507 | 0.99 (0.96-1.02) |
| Model 2 | Ref | 1.42 (0.89-2.27) | 1.15 (0.74-1.77) | 0.404 | 0.99 (0.96-1.03) |
|  |  |  |  |  |  |
| **Processed Meats** | **Low intake** | **Medium intake** | **High intake** | **p-trend** | **2 % increase** |
|  | < 14.8% | 14.8-32.1% | > 32.1% |  |  |
| **Oesophagus**, n | 82 | 65 | 46 |  |  |
| Model 1 | Ref | 0.81 (0.53-1.23) | 0.44 (0.28-0.70) | 0.001 | 0.97 (0.95-0.99) |
| Model 2 | Ref | 0.93 (0.59-1.46) | 0.53 (0.33-0.87) | 0.147 | 0.93 (0.89-0.98) |
| **Stomach**, n | 127 | 157 | 128 |  |  |
| Model 1 | Ref | 1.43 (1.01-2.00) | 0.83 (0.59-1.19) | 0.320 | 0.98 (0.97-1.00) |
| Model 2 | Ref | 1.43 (1.02-2.02) | 0.84 (0.58-1.19) | 0.286 | 0.96 (0.93-0.99) |
| **Pancreas**, n | 50 | 55 | 56 |  |  |
| Model 1 | Ref | 1.34 (0.85-2.13) | 1.01 (0.63-1.62) | 0.928 | 1.00 (0.98-1.02) |
| Model 2 | Ref | 1.37 (0.86-2.18) | 1.06 (0.66-1.72) | 0.680 | 1.00 (0.96-1.04) |
|  |  |  |  |  |  |
| **Sweets and pastries** | **Low intake** | **Medium intake** | **High intake** | **p-trend** | **2 % increase** |
|  | < 7.6% | 7.6-32.5% | > 32.5% |  |  |
| **Oesophagus**, n | 97 | 55 | 41 |  | 193 |
| Model 1 | Ref | 0.65 (0.43-0.98) | 0.50 (0.32-0.79) | 0.003 | 0.96 (0.95-0.98) |
| Model 2 | Ref | 0.71 (0.45-1.12) | 0.77 (0.47-1.24) | 0.077 | 0.98 (0.96-1.00) |
| **Stomach**, n | 104 | 156 | 152 |  | 412 |
| Model 1 | Ref | 1.56 (1.11-2.19) | 1.40 (1.00-1.96) | 0.069 | 1.01 (0.99-1.02) |
| Model 2 | Ref | 1.64 (1.15-2.32) | 1.54 (1.09-2.19) | 0.020 | 1.01 (1.00-1.02) |
| **Pancreas**, n | 51 | 51 | 59 |  | 161 |
| Model 1 | Ref | 0.99 (0.63-1.56) | 1.03 (0.66-1.62) | 0.739 | 1.00 (0.99-1.02) |
| Model 2 | Ref | 1.04 (0.65-1.65) | 1.21 (0.76-1.90) | 0.356 | 1.01 (0.99-1.03) |
|  |  |  |  |  |  |
| **UP drinks** | **Low intake** | **Medium intake** | **High intake** | **p-trend** | **2 % increase** |
|  | 0% | 0.1-32.6% | > 32.6% |  |  |
| **Oesophagus**, n | 50 | 44 | 99 |  | 193 |
| Model 1 | Ref | 1.40 (0.85-2.29) | 2.38 (1.55-3.66) | <0.001 | 1.03 (1.01-1.04) |
| Model 2 | Ref | 1.11 (0.83-2.13) | 1.33 (0.83-2.13) | 0.245 | 1.01 (1.00-1.02) |
| **Stomach**, n | 199 | 91 | 122 |  | 412 |
| Model 1 | Ref | 1.00 (0.70-1.44) | 0.98 (0.70-1.36) | 0.909 | 1.00 (0.99-1.01) |
| Model 2 | Ref | 0.93 (0.64-1.34) | 0.87 (0.62-1.22) | 0.529 | 1.00 (0.97-1.02) |
| **Pancreas**, n | 79 | 35 | 47 |  | 161 |
| Model 1 | Ref | 1.01 (0.62-1.65) | 1.00 (0.64-1.55) | 0.811 | 1.00 (0.98-1.01) |
| Model 2 | Ref | 0.88 (0.53-1.44) | 0.80 (0.50-1.27) | 0.275 | 0.99 (0.97-1.00) |
|  |  |  |  |  |  |
| **Pre-cooked food** | **Low intake** | **Medium intake** | **High intake** | **p-trend** | **2 % increase** |
|  | <5.3% | 5.3-15.3% | >15.3% |  |  |
| **Oesophagus**, n | 68 | 72 | 53 |  |  |
| Model 1 | Ref | 1.10 (0.72-1.67) | 0.79 (0.51-1.22) | 0.439 | 1.00 (0.98-1.02) |
| Model 2 | Ref | 1.16 (0.74-1.82) | 0.80 (0.50-1.29) | 0.576 | 1.00 (0.98-1.02) |
| **Stomach**, n | 149 | 133 | 130 |  |  |
| Model 1 | Ref | 0.85 (0.61-1.19) | 0.71 (0.51-1.00) | 0.043 | 0.99 (0.97-1.00) |
| Model 2 | Ref | 0.86 (0.62-1.20) | 0.70 (0.50-0.98) | 0.023 | 0.99 (0.97-1.00) |
| **Pancreas**, n | 48 | 57 | 56 |  |  |
| Model 1 | Ref | 1.10 (0.70-1.74) | 0.90 (0.57-1.42) | 0.660 | 1.00 (0.98-1.02) |
| Model 2 | Ref | 1.11 (0.70-1.75) | 0.90 (0.57-1.43) | 0.535 | 1.00 (0.97-1.02) |
|  |  |  |  |  |  |
| Model1: adjusted by age, sex and province. Model 2: Model 1 plus educational level, smoking habit, beer and wine consumption.  Abbreviations: UPF, ultra-processed food; g, grams; RRR; relative risk ratios. | | | | | |
